# Genomic, proteomic and bioinformatic analysis of two temperate phages in *Roseobacter* clade bacteria isolated from the deep-sea water

**DOI:** 10.1186/s12864-017-3886-0

**Published:** 2017-06-27

**Authors:** Kai Tang, Dan Lin, Qiang Zheng, Keshao Liu, Yujie Yang, Yu Han, Nianzhi Jiao

**Affiliations:** State Key Laboratory for Marine Environmental Science, Institute of Marine Microbes and Ecospheres, Xiamen University, Xiamen, 361102 People’s Republic of China

**Keywords:** Marine phage, Genomics, Proteomics, Phylogenetic analysis, Roseobacter clade bacteria

## Abstract

**Background:**

Marine phages are spectacularly diverse in nature. Dozens of roseophages infecting members of *Roseobacter* clade bacteria were isolated and characterized, exhibiting a very high degree of genetic diversity. In the present study, the induction of two temperate bacteriophages, namely, vB_ThpS-P1 and vB_PeaS-P1, was performed in *Roseobacter* clade bacteria isolated from the deep-sea water, *Thiobacimonas profunda* JLT2016 and *Pelagibaca abyssi* JLT2014, respectively. Two novel phages in morphological, genomic and proteomic features were presented, and their phylogeny and evolutionary relationships were explored by bioinformatic analysis.

**Results:**

Electron microscopy showed that the morphology of the two phages were similar to that of siphoviruses. Genome sequencing indicated that the two phages were similar in size, organization, and content, thereby suggesting that these shared a common ancestor. Despite the presence of Mu-like phage head genes, the phages are more closely related to *Rhodobacter* phage RC1 than Mu phages in terms of gene content and sequence similarity. Based on comparative genomic and phylogenetic analysis, we propose a Mu-like head phage group to allow for the inclusion of Mu-like phages and two newly phages. The sequences of the Mu-like head phage group were widespread, occurring in each investigated metagenomes. Furthermore, the horizontal exchange of genetic material within the Mu-like head phage group might have involved a gene that was associated with phage phenotypic characteristics.

**Conclusions:**

This study is the first report on the complete genome sequences of temperate phages that infect deep-sea roseobacters, belonging to the Mu-like head phage group. The Mu-like head phage group might represent a small but ubiquitous fraction of marine viral diversity.

**Electronic supplementary material:**

The online version of this article (doi:10.1186/s12864-017-3886-0) contains supplementary material, which is available to authorized users.

## Background

Marine phages are one of the most abundant biological components of marine environments and are believed to significantly contribute to the microbial loop and biogeochemical cycles of the ocean [1–3]. Although the emergence of cultivation-independent tools such as metagenomics have expanded our understanding of viral community composition and their genetic diversity [4], research studies in the field of marine virology have focused on identification of phages infecting ecologically important environmental bacteria [5]. The importance of phage isolation is exemplified by studies on phages infecting the ubiquitous marine bacteria such as cyanophages of *Cyanobacteria* [6], SAR11 clade viruses [7], and roseophages of *Roseobacter* clade bacteria (RCB) [8].

RCB are globally distributed throughout the surface oceans and involved in biogeochemical transformations [9]. All members of the RCB cluster belong to the *Rhodobacteraceae* family of *Alphaproteobacteria* and constitute up to 25% of all marine microbial communities [10]. Virus-mediated gene transfer is considered one of the most important factors that influence RCB genomic diversity and ecological adaptation [9]. Dozens of phages infecting RCB are isolated and sequenced, including those of roseophages SIO1 [11, 12], DSS3Φ2 [8], EE36Φ1 [8],ΦCB2047-B [13, 14], RDJLΦ1 [15, 16], P12053L [17], RPP1 and RLP1 [18], and vB_DshP-R1 [19, 20]. Among the known RCB strains, *Roseovarius nubinhibens* ISM and *Silicibacter* sp. TM1040 harbor one and three mitomycin C-inducible prophages, respectively [21, 22].

Recent viral ecological studies have indicated that phages provide an important, yet previously ignored contribution to deep-sea ecosystems functioning and environmental adaptation to its hosts [3, 23–25]. Compared to viruses in coastal and estuarine environments, lysogeny seems to be more prevalent in the deep biosphere, as indicated by the presence of high amounts of temperate phages [25]. Although attempts to isolate phages from deep-sea bacteria have been successful in several cases [26–28], these remain largely unexplored because only a few hosts have been cultivated. To date, our understanding of deep-sea roseobacter phages is limited. *Pelagibaca abyssi* JLT2014 [29] and *Thiobacimonas profunda* JLT2016 [30] are two RCB members that have been isolated from the deep-seawater (water depth: 2000 m and 2571 m) of the Southeastern Pacific Ocean. A recent study suggests that two deep-sea roseobacter bacteria have mixotrophic capacities that these may be potentially utilized in chemolithotrophic carbon dioxide fixation [31].

The present study characterized phages of deep-sea roseobacters by DNA sequencing and proteomics analysis, resulting in the identification of two mitomycin C-induced temperate phages that contain Mu-like elements and transposases, hereby designated as *Thiobacimonas* phage vB_ThpS-P1 and *Pelagibaca* phage vB_PeaS-P1. Mu-like bacteriophages are phylogenetically related to Mu phages and have been isolated primarily from *Gammaproteobacteria* such as *Escherichia* phage D108 and *Haemophilus* phage SuMu belonging to the *Myoviridae* family [32, 33], and *Pseudomonas* phages D3112 and B3 affiliated to the *Siphoviridae* family [34, 35]. The Mu-like phage RcapMu with siphovirus-like morphology was induced using high temperature from *Rhodobacter capsulatus* SB1003, which belongs to the *Rhodobacteraceae* family [36] and is the first reported transposing bacteriophage that infects *Alphaproteobacteria*. Mu-like prophages are generally not inducible by mitomycin C [32]. However, lysogenic phage vB_CibM-P1 with Mu-like elements was induced by mitomycin C from *Citromicrobium* sp. JLT354 within marine *Alphaproteobacteria,* and it showed a myovirus-like morphology [37]. Two novel phages in roseobacters, vB_ThpS-P1 and vB_PeaS-P1, contain structural modules and proteomes similar to those of the Mu and Mu-like phages. However, unlike Mu and Mu-like phages, these are incapable of carrying variable amounts of host DNA during both lytic and lysogenic development. The present study compared phages containing Mu-like elements to typical Mu and Mu-like phages, herein designated as the “Mu-like head phage group”, to resolve discrepancies between function and phylogeny of transposable phages.

## Methods

### Phage induction

*P. abyssi* JLT2014 and *T. profunda* JLT2016 were cultured in rich organic medium (1 g yeast extract, 1 g Bacto-peptone, and 1 g sodium acetate per liter of artificial seawater with vitamins and trace elements) at 28 °C at a constant rotation of 160 rpm. The induction process and sampling were performed as earlier described [21, 37]. Briefly, bacterial suspensions were cultured in two 500-mL conical flasks until these reached a stable growth phase; mitomycin C (final concentration: 0.5 μg/mL) was added to one, whereas the other served as the control. After mitomycin C treatment for 30 min, the cells in both the control and treatment tubes were centrifuged, washed, and resuspended in 500 mL of fresh rich organic medium. Samples (2 mL) for viral and bacterial counting were immediately fixed with glutaraldehyde (final concentration: 1%) for 15–20 min in the dark and then stored in an −80 °C refrigerator for flow cytometry analysis. Virus counting was conducted using an Epics Altra II flow cytometer (Beckman-Coulter, USA), and bacterial counts were determined by using a BD Accuri C6 flow cytometer. Samples were diluted in 0.2-μm filtered TE buffer (Tris-EDTA, pH 8), and stained with the DNA dye SYBR Green I (Molecular Probes, Inc., USA). The bacterial and viral particles were identified and counted as described elsewhere [38, 39]. All reagents used in the experiments were obtained from Sigma-Aldrich (USA) unless otherwise specified.

### Phage purification

Phage particles in lysates were harvested and purified as previously described [21, 37]. Phage lysates were treated with RNase A (final concentration: 2 μg/mL) and DNase I (final concentration: 2 μg/mL) by incubating for 1 h and then centrifuging at 10,947×g for 10 min in a Thermo Scientific Sorvall ST-16R. Supernatants were filtered through a 0.45-μm pore size filter (type HA, Millipore, USA) to remove host cells and cellular debris. Phage particles in the filtrate were treated with polyethylene glycol 8000 (final concentration: 100 g/L) overnight at 4 °C and precipitated by centrifugation at 10,947×g for 60 min. The pellets were resuspended in 6 mL of SM buffer (10 mM NaCl, 50 mM Tris, 10 mM MgSO _4_ , and 0.1% gelatin) and then incubated overnight at 4 °C. The phage suspension was mixed with CsCl (final concentration: 0.6 g/mL) and centrifuged in an Optima™ L-100XP (200,000×g for 24 h at 4 °C). Visible bands were extracted and then dialyzed (molecular weight: 530 kDa) twice in SM buffer overnight at 4 °C.

### Transmission electron microscopy for phage morphology

One drop of purified phage suspension was adsorbed to a Formvar/carbon-coated 200-mesh copper grid for 10 min and negatively stained with 2% (wt/L) phosphotungstic acid in the dark for 30 s. After 30 min of drying, the grid was examined using a JEM-2100 transmission electron microscope (JEOL, Japan) or a Tecnai G2 Spirit transmission electron microscope at 120 KeV (Thermo Fisher Scientific, USA). Images were captured using a GATAN INC CCD image transmission system.

### DNA preparation and genome sequencing

Phage DNA was extracted as described elsewhere [21], dissolved in TE buffer (10 mM Tris, 1 mM EDTA), and stored at 4 °C. DNA library preparation was performed according to the NEBNext® Ultra™ DNA Library Prep Kit for Illumina (NEB, USA). Approximately 10 ng of the DNA sequencing library was used to generate a cluster in cBot using a TruSeq PE Cluster Kit (Illumina, USA) and then sequenced in an Illumina HiSeq™ 2500 system for 2 × 125 bp data. The raw data were filtered using a FASTX-Toolkit to remove the adapters, N bases, and low-quality reads (http://hannonlab.cshl.edu/fastx_toolkit/). Clean reads were mapped to the bacterial complete genome sequences [31] using Bowtie 2 [40], and then two prophage regions with an average coverage of 8000× and 7500× were compared to the whole genome, which had a depth of about 10× and 15×, respectively.

The sequences of phages vB_ThpS-P1 and vB_PeaS-P1 have been deposited in the GenBank database under Accession Number KT381864 and KT381865, respectively.

### Proteomics analysis

The purified phages were treated with a lysis buffer (1 mM EDTA, 250 mM Tris-HCI (pH 6.8), 4% 2-mercaptoethanol, 4% SDS, 50% glycerol, and 0.02% bromphenol blue) at 100 °C for 10 min, and stored at −20 °C for further protein analysis. Protein in-solution digestion was performed according to the FASP procedure [41]. Proteomics analysis was performed on a Q Exactive mass spectrometer that was coupled to an Easy nLC (Thermo Fisher Scientific, USA). The instrument was run with the peptide recognition mode enabled. MS/MS spectra were searched using a MASCOT engine (Matrix Science, London, UK; version 2.2) against phage genomes. For protein identification, the following options were used. Peptide mass tolerance = 20 ppm, MS/MS tolerance = 0.1 Da, enzyme = trypsin, missed cleavage = 2, fixed modification: carbamidomethyl (C), and variable modification: oxidation (M).

### Bioinformatics analysis

Clean high-depth mapped datasets were assembled using Velvet (v1.2.03) [42]. The final assembled phage genome was automatically annotated, then manually corrected through the RAST server using SEED annotation tools [43]. Prophage-like sequences at the gene cluster level in NCBI GenBank bacterial genomes (01/2015) were detected by using a MultiGeneBlast (v1.1.14) architecture search with vB_ThpS-P1, vB_PeaS-P1, and vB_CibM-P1 genomes as queries [44]. The prophage identification tool PHAge Search Tool (PHAST) was used to determine the region containing prophage like elements in bacterial genomes [45]. The clustering of sequences into homologous families was performed using SiLiX (v1.2.8) using a minimum identity threshold of 80% and default values for the remaining parameters [46]. The gene content of phages and phage-like elements were visualized by a hierarchical clustering method using Gene-E tool (https://software.broadinstitute.org/GENE-E/download.html). Phylogenetic trees were based on maximum-likelihood and neighbor-joining methods and constructed using MEGA 6.0with a JTT model, with gamma set to 4 [47]. Bootstrap resampling was performed for 1000 replications. BLAST-based average nucleotide identity was determined using JSpecies (v1.2.1) [48]. An InterPro database search of *gp*T and *gp*23 genes sequences in the *Tara* Oceans metagenomic datasets was performed with the protein domain IPR018774 and IPR010762 as queries (https://www.ebi.ac.uk/metagenomics/projects/ERP001736) [49, 50]. To remove bias to the average genome size with gene sampling of genes from a given metagenomic community and the effects of gene size on hit retrieval, the abundance of *gpT* gene relative to the number of single-copy genes (*recA*) hits for each site was calculated as previously described [51], which was as follows: Number of single-copy genes (*recA*) = Number of size-normalized *gpT* gene hits/Average number of *recA* gene hits. All sequences in NCBI viral genomes and Mu-like head phages were Blast against the Pacific Ocean Virome, respectively (E-value 0.00001) [52].

## Results and Discussion

### Phage Induction and Morphology

**Fig. 1:**
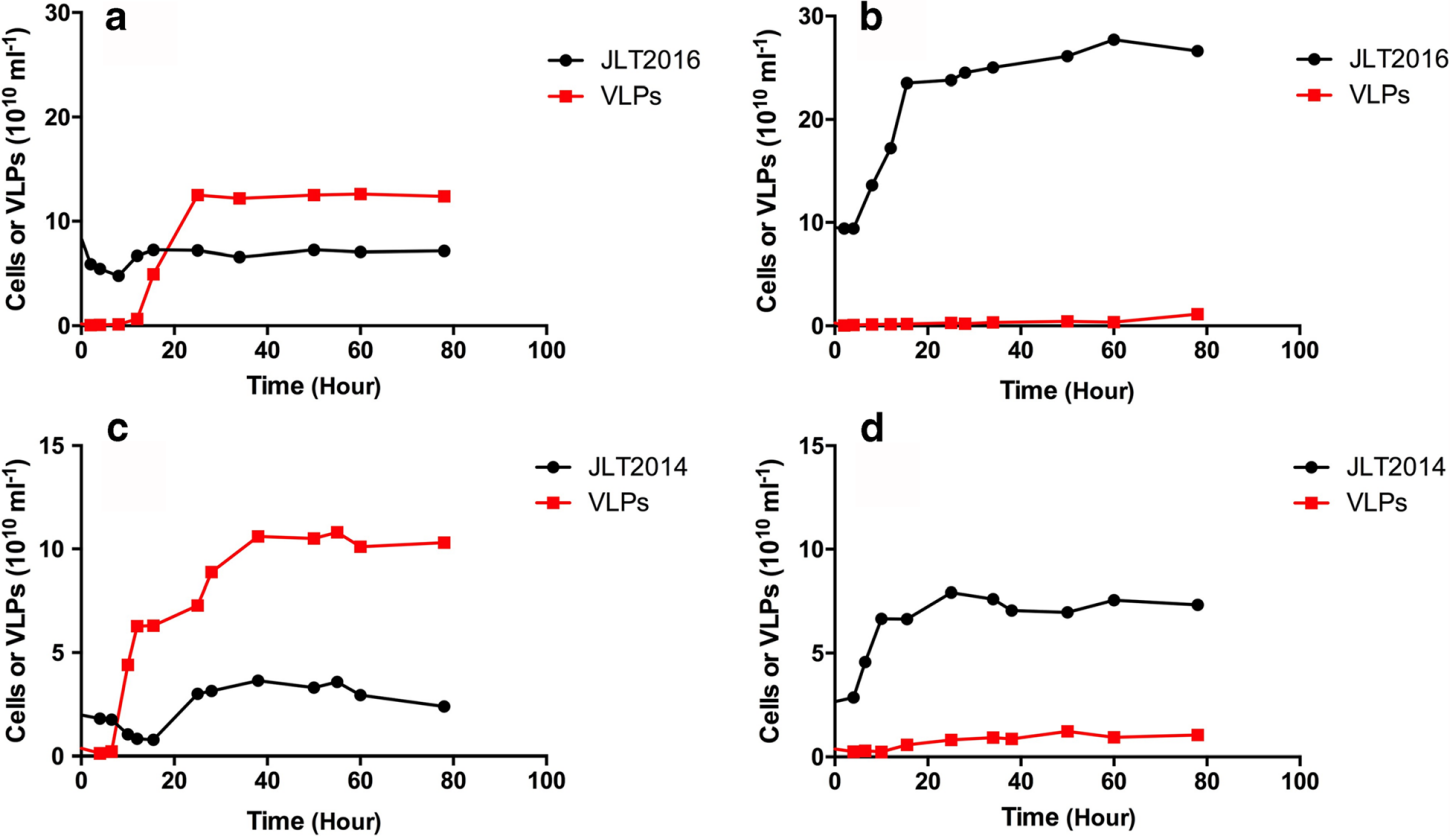
Viral particle yield following mitomycin C induction of *T. profunda* JLT2016 and *P. abyssi* JLT2014. Flow cytometry counts of JLT2016 and JLT2014 cells and viral-like particles were performed with **a**, **c** a mitomycin C-treated culture and **b**, **d** a control culture without mitomycin C

**Fig. 2:**
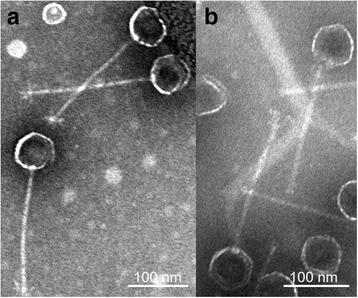
Electron micrographs of purified phage vB_ThpS-P1 **a** and vB_PeaS-P1 **b** particles with a typical siphovirus-like morphology. Scale bar: 100 nm

*T. profunda* JLT2016 growth was apparently inhibited after the addition of mitomycin C at the exponential growth phase, whereas that of virus-like particles (VLPs) rapidly increased to 4.9 × 10^10^ particles/mL after 15 h (Fig. 1A and B). A dramatic increase in the number of inducible VLPs (4.4 × 10^10^ particles/mL) in *P. abyssi* JLT2014 was observed within 10 h of mitomycin C treatment (Fig. 1C and D). The two transposable prophages were not induced at a high temperature (42 °C), whereas the transposable coliphage Mu is usually induced by high temperature rather than mitomycin C [32]. When these induced VLPs were used to re-infect two strains, lytic interactions between the phages and strains were not observed. A lysogenic bacterium is resistant to reinfection by the same or related phages because an “immunity” is conferred by the presence of the prophage [53]. The induced bacteriophage vB_ThpS-P1 exhibited a siphovirus-like morphology with a long flexible and non-contractile tail (Fig. 2A). The average particle had a head size of approximately 63 ± 3 nm and tail length of approximately 205 ± 4 nm. The inducible phage vB_PeaS-P1 was morphologically identical to that of vB_ThpS-P1 phages, with only a slight difference in length and width (head size: 64 ± 2 nm; and tail length: 211 ± 3 nm; Fig. 2B). The tail features of the two deep-sea roseobacters phages were similar to those of the well-characterized Mu-like phage RcapMu [36].

### Genomic Features

vB_ThpS-P1 contained double-stranded (ds) DNA of 39,591 bp in size and a GC content of 66.7%, which was similar to that of the complete genome of *T. profunda* JLT2016 (67.1%) [31]. The phage vB_PeaS-P1 genome consisted of 38,686 bp of dsDNA and a GC content of 63.8%, which was similar to that of its host DNA (66%) [31]. No tRNA sequences were detected in their genomes.

**Fig. 3:**
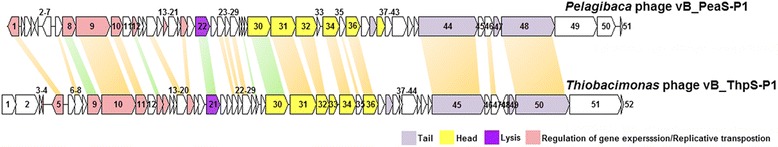
Genomic maps of vB_ThpS-P1 and vB_PeaS-P1. ORFs are color-coded according to predicted function: thistle, tail; yellow, head; dark violet, lysis; light pink, regulation of gene expression/replicative transposition; white, hypothetical proteins. The numbers in each box are the ORF numbers, which correspond to those used in the text and table. The relatively high homologous sequences are indicated by green shading (amino acid identity >50%), and other homologs are indicated by orange shading

A total of 52 and 51 open reading frames (ORFs) were identified in vB_ThpS-P1 and vB_PeaS-P1, respectively. A total of 24 ORFs and 19 ORFs were functionally annotated in vB_ThpS-P1 and vB_PeaS-P1 genomes, respectively (Table 1). ORFs in the genomes of the two phages were mostly oriented in a single direction, whereas two phage repressor genes (ORF5 in vB_ThpS-P1 and ORF1 in vB_PeaS-P1) were transcribed in the reverse direction (Fig. 3). They showed almost the same functional genes, except for phage vB_ThpS-P1 lacked the gene encoding for DNA-binding protein HU-beta. These also harbored Mu-like phage *gp36* and *gp29* genes, which might play a role in virulence [33]. The phage genomes both possessed ParB homologs and two putative transposase genes, which might function in phage integration into the host genome [54]. Each phage genome contained a repressor (Cro/CI family) gene, which prevents transcription and translation of lysis and other late genes [27]. These genes, which act as a lysogeny module, are common features of temperate phages. Based on the putative functions of ORFs, the genome of vB_ThpS-P1 and vB_PeaS-P1 shared a similar backbone that could be roughly divided into four functional modules (Fig. 3): the right half largely encodes functions required for structure and lysis (putative phage gene expression late region: tail, head, and lysis modules), whereas the left half mainly encodes proteins that modulate phage gene expression or host response and lysogeny (putative phage gene expression in the early or middle region). The genomes of the two phages were distinct from that of other known roseophages [11–20].

**Table 1:** Genomic and proteomic features of phages vB_ThpS-P1 and vB_PeaS-P1

ORF	Annotation	Peptides^a^	Unique Peptides^a^	Homologs^b^	ORF	Annotation	Peptides^a^	Unique Peptides^a^	Homologs^b^
*Thiobacimonas*phage vB_ThpS-P1 (size:39,591 bp; GC content:66.7%)					*Pelagibaca* phage vB_PeaS-P1 (size:38,686 bp; GC content:63.8%)				
ORF1	Hypothetical protein			0	ORF1	Phage repressor			9
ORF2	Hypothetical protein			0	ORF2	Hypothetical protein			6
ORF3	Hypothetical protein			0	ORF3	Hypothetical protein			1
ORF4	Hypothetical protein			0	ORF4	Hypothetical protein			0
ORF5	Phage repressor			9	ORF5	Hypothetical protein			0
ORF6	Hypothetical protein			0	ORF6	Hypothetical protein			0
ORF7	Hypothetical protein			0	ORF7	Hypothetical protein			0
ORF8	Hypothetical protein			2	ORF8	Chromosome partitioning protein parB			66
ORF9	Chromosome partitioning protein parB			66	ORF9	Mu-like phage Flu Mu transposase A			189
ORF10	Mu-like phage Flu Mu transposase A			189	ORF10	Mu-like phage Flu Mu transposase B	6	6	128
ORF11	Mu-like phage Flu Mu transposase B			128	ORF11	Hypothetical protein			109
ORF12	Hypothetical protein			8	ORF12	Transcriptional regulator			146
ORF13	Transcriptional regulator			146	ORF13	Hypothetical protein			0
ORF14	DNA transposition protein gpB			0	ORF14	Hypothetical protein			2
ORF15	Hypothetical protein			0	ORF15	Protein of unknown function DUF3164	2	2	363
ORF16	Hypothetical protein			0	ORF16	Hypothetical protein			0
ORF17	Protein of unknown function DUF3164			363	ORF17	DNA-binding protein HU-beta			96
ORF18	Mu-like phage transcriptional regulator			610	ORF18	Hypothetical protein			0
ORF19	Hypothetical protein			22	ORF19	Hypothetical protein			1
ORF20	Hypothetical protein			13	ORF20	Mu-like phage transcriptional regulator			610
ORF21	N-acetylmuramoyl-L-alanine amidase	2	2	12	ORF21	Hypothetical protein			0
ORF22	Hypothetical protein			1	ORF22	N-acetylmuramoyl-L-alanine amidase	6	5	12
ORF23	Hypothetical protein			0	ORF23	Hypothetical protein			0
ORF24	Hypothetical protein			15	ORF24	Hypothetical protein			0
ORF25	Mu-like phage FluMu protein gp26	3	2	493	ORF25	Mu-like phage FluMu protein gp26	6	5	493
ORF26	Hypothetical protein			16	ORF26	Hypothetical protein			16
ORF27	Hypothetical protein			1	ORF27	Hypothetical protein			2
ORF28	Hypothetical protein			1	ORF28	Hypothetical protein			1
ORF29	Hypothetical protein			0	ORF29	Hypothetical protein			0
ORF30	Mu-like phage FluMu protein gp28			494	ORF30	Mu-like phage FluMu protein gp28			494
ORF31	Mu-like phage FluMu protein gp29	122	28	564	ORF31	Mu-like phage FluMu protein gp29	42	30	564
ORF32	Mu-like phage FluMu F protein	53	15	635	ORF32	Mu-like phage FluMu F protein	4	3	2
ORF33	Virion morphogenesis protein	1	1	683	ORF33	Hypothetical protein	4	2	0
ORF34	Mu-like phage I protein	124	15	552	ORF34	Mu-like phage I protein	2	2	552
ORF35	Hypothetical protein	86	5	253	ORF35	Hypothetical protein	90	8	253
ORF36	Mu-like phage major head subunit gpT	172	20	537	ORF36	Mu-like phage major head subunit gpT	197	36	537
ORF37	Hypothetical protein			1	ORF37	Hypothetical protein			1
ORF38	Mu-like phage FluMu protein gp36	51	9	675	ORF38	Mu-like phage FluMu protein gp36	11	8	675
ORF39	Hypothetical protein	26	7	88	ORF39	Virion morphogenesis protein			683
ORF40	Hypothetical protein			0	ORF40	Hypothetical protein	7	6	3
ORF41	Hypothetical protein	65	10	49	ORF41	Hypothetical protein	80	18	3
ORF42	Hypothetical protein			0	ORF42	Hypothetical protein			17
ORF43	Hypothetical protein			0	ORF43	Hypothetical protein			3
ORF44	Hypothetical protein			23	ORF44	Tail protein	46	39	4
ORF45	Tail protein	84	34	0	ORF45	Hypothetical protein	2	2	0
ORF46	Hypothetical protein	11	3	13	ORF46	Hypothetical protein	2	2	12
ORF47	Hypothetical protein	20	6	12	ORF47	Tail protein			13
ORF48	Hypothetical protein			0	ORF48	Tail protein	24	20	13
ORF49	Tail protein			13	ORF49	Hypothetical protein	60	34	0
ORF50	Tail protein	41	18	13	ORF50	Hypothetical protein			0
ORF51	Hypothetical protein	57	15	0	ORF51	Hypothetical protein			0
ORF52	Hypothetical protein			0					

^a^The number of and peptide and unique peptide detected from tandem mass spectrometry (MS/MS) are list

^b^The number of identified sequence homologous to two phages from bacterial genomes containing a set of Mu-like elements (Additional file 4: Table S4)

**Fig. 4:**
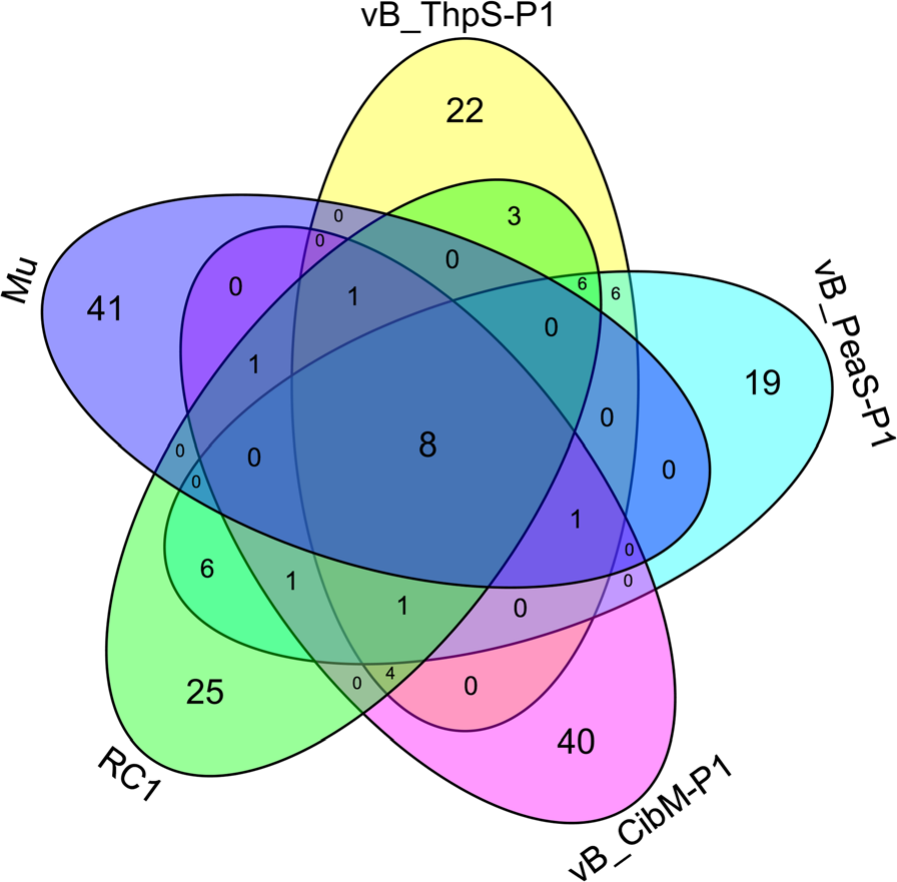
Venn diagram of vB_ThpS-P1, vB_PeaS-P1, RC1, and Mu in relation to homologous gene families. Venn diagram comparing homologous gene families in vB_ThpS-P1, vB_PeaS-P1, RC1, and Mu

The genomes of vB_ThpS-P1 and vB_PeaS-P1 had Mu-like phage homologs, which include the head morphogenesis and transposases genes. Although the modular organization and gene content of its structural head module were similar to that of Mu-like phages, some differences between its genomes and that of the Mu phage were identified. The two phages shared more sequence homologs with a *Rhodobacter* phage RC1 (23 homologs between vB_ThpS-P1 and RC1, 22 homologs between vB_PeaS-P1 and RC1) than with the Mu or RcapMu phages, suggesting that the two phages were closely related to RC1 at the genomic level (Fig. 4). *Rhodobacter* phage RC1 was induced from *Rhodobacter*sp. E32, which was isolated from the deep-sea sediment (water depth: 5086 m) and belonged to the *Siphoviridae* family (GenBank Accession NumberNC_020839.1). The two phages also lacked the host-nuclease inhibitor protein, Gam, and the Mor transcription activator, which are high-frequency proteins that exist in Mu and Mu-like phages [55], whereas the two novel phages have the ParB protein, which was not detected in the Mu phage. In addition, the virus-like particle did not harbor any random host-derived sequences (>1 kb) at its genomic DNA termini, which is a unique feature of Mu phage-related phages [32, 34].

### Proteomic features

A detailed proteomic characterization of virion particles by using high-resolution LC-mass spectrometry identified 15 and 18 proteins in vB_ThpS-P1 and vB_PeaS-P1, respectively (Table 1). The same functional proteins were identified in the two phages, including one tail protein, four Mu-like head structural proteins, one Mu-like phage gp26 protein, one lysis-related protein, and two hypothetical proteins. Based on mass spectrometry spectral count, the most abundant structural protein detected in both phages was the major head protein, Mu-like phage gpT protein. Other proteins that predominated in both phages were encoded in the genomic tail and morphogenesis modules. Mu-like phage gp36 protein and gp29 protein were identified in both. Of the hypothetical proteins in the two phages, an unknown protein with a DUF3164 domain was detected. A transposase B protein was detected in vB_ThpS-P1. A transposase B protein normally promotes efficient transposition and is directly involved in the choice of DNA target sites and immunity to self-integration [56, 57].

### Phylogenetic analysis

**Fig. 5:**
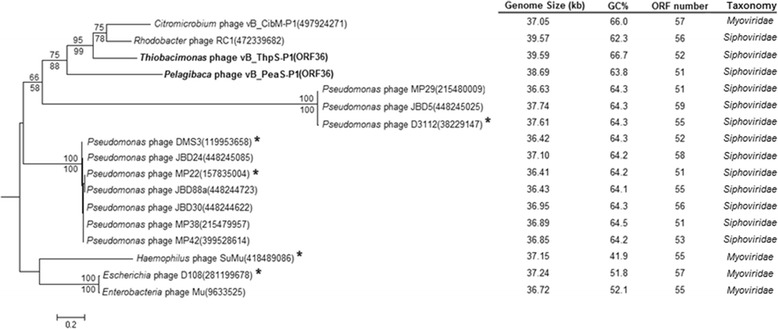
Phylogenetic analysis based on amino acid sequences associated with major head subunit proteins. Mu- and Mu-like phages are indicated byasterisks. The bootstrap values of maximum likelihood (above) and neighbor-joining (below) methodsare shown in the tree. The scale bar represents 0.2 fixed mutations per amino acid position. The numbers in brackets represent the corresponding GenBank ID. The summary of genome sizes (exclusive of the length of host random DNA in a Mu-like phage), GC contents, and predicted ORFs of (pro)phages are shown on the right side of the phylogenetic tree

Phylogenetic analysis based on the conserved amino acid sequences of the Mu-like phage gpT protein supported the finding that among all the known phages but distinct from previously characterized Mu and Mu-like, vB_ThpS-P1 and vB_PeaS-P1 were most closely related to RC1 (Fig. 5). This tree resolved one subgrouping of the siphovirus phages, including the known RC1 and *Pseudomonas* phages and one subgrouping of the myovirus phages, including Mu-like phage SuMu [33] and Mu phage [32]. However, this tree placed the Mu-like phage gpT protein of myovirus-like phage vB_CibM-P1 [37] in the clade that included RC1, vB_ThpS-P1, and vB_PeaS-P1. In addition, their similar phylogenetic relationships were recovered with the reconstruction of other five other head structural proteins (Additional file 1: Fig. S1), transposase A or B, and transcriptional regulator proteins, respectively (Additional file 1: Fig. S2). To reconcile possible discrepancies between phylogeny and morphologyof transposable phages, a new family *Saltoviridae* of the order *Caudovirales* was recently proposed, which included subfamilies *Myosaltovirinae* and *Siphosaltovirinae* [58]. Phylogenetic analysis combined with morphological assessment indicated that the two phages could be taxonomically classified into the *Siphosaltovirinae*. The myovirus genomes have a notably lower GC content than the siphoviruses, with the exception of vB_CibM-P1. Furthermore, these have similar genome sizes and number of ORFs (Fig. 5).

### Distribution of the Mu-like phage elements in bacterial genomes

**Fig. 6:**
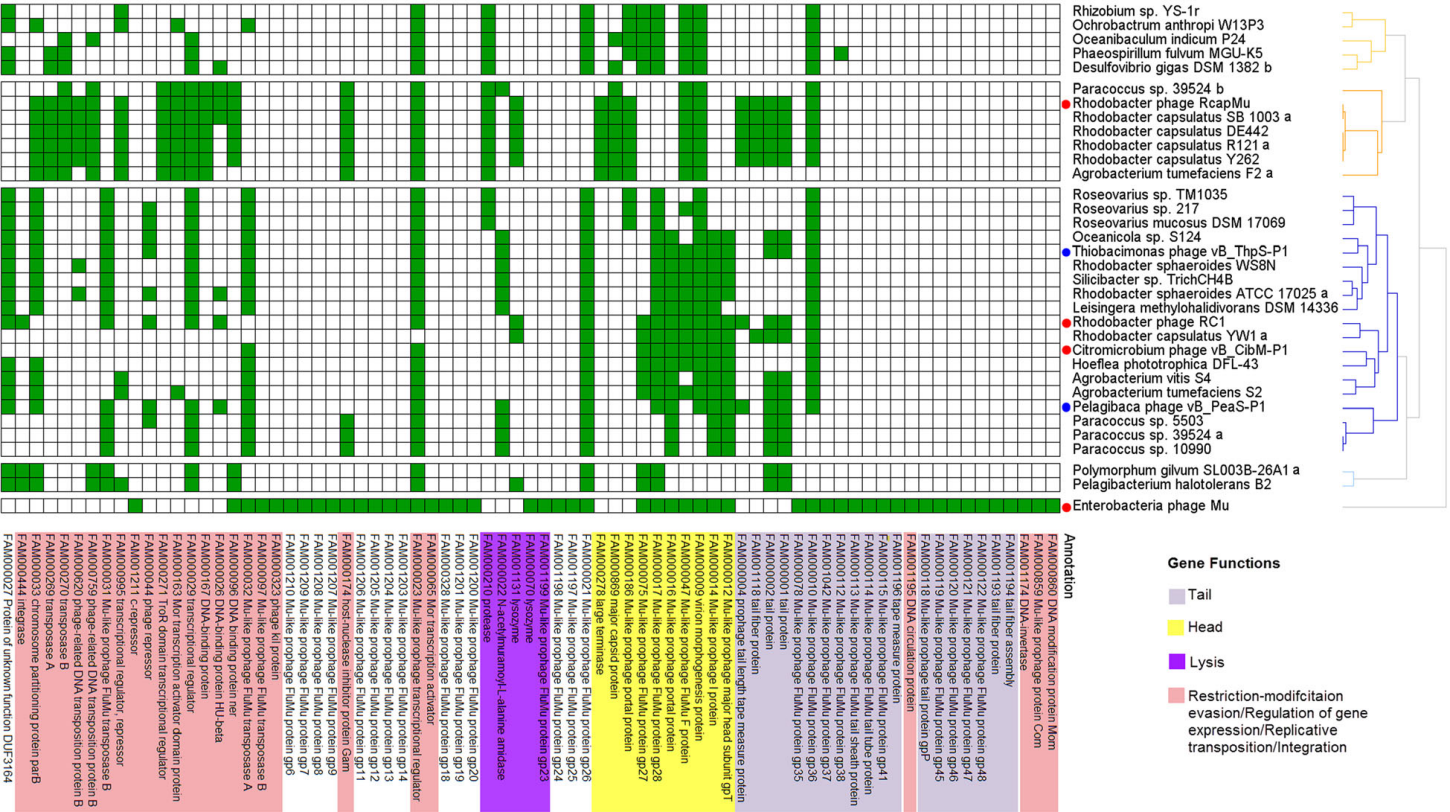
Hierarchical clustering of vB_ThpS-P1, vB_PeaS-P1, and prophage-like elements among bacteria in terms of gene content, which was based on SiliX homology clustering. *Green* represents the presence of a gene. Two prophages in this study were labeled with *blue* dots and other reference phages are labeled as red dots. ORFs are color-coded according to predicted function: thistle, tail; *yellow*, head; *dark violet*, lysis; *light pink*, restriction-modification evasion/regulation of gene expression/replicative transposition/integration; white, hypothetical proteins. For the complete hierarchical clustering map, please refer to Additional file 5: Table S4

Mu-like phage elements occur in more than 130 bacterial genera (Additional file 2: Table S1). The predicted Mu-like phage regions are not only found in species of *Escherichia*, *Vibiro*, *Haemophilus*, *Burkholderia*, *Neisseria*, *Pseudomonas*, and *Rhodobacter*, in which Mu-like prophages were described [32–36, 59–62], but also in unexplored species such as strains of RCB (Additional file 2: Table S1). Most of the identified phage elements contain the Mu-like phage head genes, which suggest that head structural genes in Mu-like elements are relatively stable at the genomic level (Additional file 2: Table S1). These have at least one transposase and one transcriptional regulator (Additional file 3: Table S2). Prophage-like elements frequently harbor the DUF3164 and Mu-like phage gp26 proteins (Table 1). Based on the gene content of phage and phage-like elements, the Mu and RcapMu phages were classified into a separate branch from that of the clade containing phages vB_ThpS-P1, vB_PeaS-P1, RC1, and vB_CibM-P1 (Fig. 6). We proposed a “Mu-like head phage group” allowing the inclusion of phages that contain Mu-like head structural genes. The phages vB_ThpS-P1, vB_PeaS-P1, RC1, and vB_CibM-P1 were classified as members of the Mu-like head phage group, which reflects its evolutionary relationship with known Mu-like phages that, in turn, could be included in the group. For instance, Mu-like head phages infecting *P. aeruginosa* contain Mu-like phages (such as D3112, DMS3, and MP22) and the other transposable phages (Fig. 5). Their genomes are similar and share nearly identical Mu-like head proteins (Additional file 1: Figure S1).

### Geographic distribution of members of the mu-like head phage group

Hosts of phage vB_CibM-P1 were obtained from a seawater depth of 75 m [37], whereas hosts of vB_ThpS-P1 and vB_PeaS-P1 were isolated from two distinct stations at depths of 2000 m and 2571 m in the bathypelagic water, respectively [29, 30]. The average relative abundances of *gpT* genes of Mu-like phages in the *Tara* ocean metagenomic datasets for surface and mesopelagic water samples were 1.3% and 0.4%, respectively, with an average ratio of 1% between the *gpT* genes of Mu-like phages and *gp23* genes of T4-like bacteriophages (Additional file 4: Table S3), implying that the hosts of Mu-like head prophages rarely occur in the surface water down to the mesopelagic zone. The highest relative abundance of *gpT* genes (10.0%) was observed in one station of the South Atlantic at a depth of 800 m (Additional file 4: Table S3). The sequences of Mu-like head phage group were represented in 0.4% of the deep-sea viral metagenomesin the Pacific Ocean. A Mu-like head phage *Rhizobium* phage RR1-B was found to significantly contribute to temperate phages abundance in the deep subseafloor sediments [23]. These analyses revealed that this phage group is distributed ubiquitously in the marine environment.

### Evolutionary implications for the mu-like head phage group

The vB_ThpS-P1 and vB_PeaS-P1 genomes exhibited an average nucleotide identity (ANIb) of 65.1% (69% genome involved in alignment), which was derived from random fragment BLAST, whereas the ANIb value of vB_ThpS-P1 and RC1, vB_PeaS-P1 and RC1 was 62.9% and 63.1%, respectively (both >60% genome alignment). Their low level of nucleotide identity and the observation that only five pairs of homologous sequences showing >50% amino acid identity (Fig. 3) suggest that a significant number of mutations might have occurred in the phage genome, similar to that observed in other tail bacteriophages [63]. In addition, the genomes of the novel phages in the two roseobacters exhibited a mosaic relationship with the other phages (Additional file 5: Table S4), which was similar to other dsDNA tailed bacteriophages [63, 64].

Several *Siphoviridae*-like tail genes in vB_ThpS-P1, vB_PeaS-P1 and RC1 have homologous relationships with each other (Fig. 6). Majority of head ORFs in vB_CibM-P1 possess a close phylogenetic relationship with those in RC1 (Fig. 5 and Additional file 1: Figure S1). Furthermore, they were closely clustered with RC1 based on gene content (Fig. 6). However, vB_CibM-P1 has a mosaic genomic structure that includes a *Myoviridae*-like tail and a *Siphoviridae*-like head. One tail protein of vB_CibM-P1 showed a low amino acid identity with the corresponding *Rhizobium* phage protein [37]. Based on these observations, we hypothesized that an ancestor of vB_CibM-P1 might belong to siphovirus, and their tail genes were subsequently replaced with those of other phages through the horizontal exchange of genetic material.

On the other hand, domestication of prophages by bacteria can result in the loss of substantial amounts of genes in the host genome [65]. Most of the identified prophage regions in bacterial genomes (Additional file 2: Table S1) are more likely to be prophage remnants because its genomic length and number of ORFs were respectively shorter and lower than those of the characterized Mu-like head phages (Fig. 5). A Mu-like phage remnant in the RCB strain *Oceanicola* sp. S124 harbored 27 ORFs that were homologous to vB_ThpS-P1, which was indicative of a close evolutionary relationship (Fig. 6). The prophage remnants were identified in other RCB genera, including *Phaeobacter*, *Roseovarius*, *Hoeflea*, *Silicbacter*, *Leisingera*, and *Ruegeria* (Additional file 2: Table S1). Several lysogenic genes or head structural genes were identified in *Citreicella* sp. SE45 and *Roseobacter denitrificans* OCh114 (Additional file 2: Table S1). Thus, genetic mutation, gene acquisition and gene loss might contribute to the diversity of this phage group.

## Conclusions

This study showed that two novel phages in deep-sea roseobacters have similar morphological, genomic and proteomic features. Based on gene content and phylogenetic analysis, we proposed a “Mu-like head phage group” allowing the inclusion of two novel phages, Mu-like phages and others that all contained homologous head elements sequences, to reconcile a significant number of discrepancies function and phylogeny of transposable phages. The Mu-like head phage group sequences are found to be common and widely distributed in the marine environment. Further work will need to explore the ecological role of this group in nature. The novel phages and roseobacters from this study provide phage–host systems for biological hypotheses testing.

## Additional files

Additional file 1: Figure S1. Phylogenetic trees of the head modules proteins of (pro)phages. Maximum likelihood tree and neighbor-joining tree methods and bootstrap analysis (100 replicates) based on the alignment of the amino acid sequence of the I protein (A), the virion morphogenesis protein (B), the Mu-like phage F protein (C), the Mu-like phage gp29 protein (D), and the Mu-like phage gp28 protein (E) of (pro)phages. The numbers at the nodes indicate bootstrap probabilities of that particular branch of the maximum likelihood (above) and neighbor-joining (below) trees. **Figure S2.** Phylogenetic trees of the transcriptional regulator (A), transposase B (B), and transposase A (C) of the (pro)phages. The numbers at the nodes indicate the bootstrap probabilities of that particular branch of the maximum likelihood (above) and neighbor joining (below) trees. (PDF 191 kb)

Additional file 2: Table S1. The identified regions containing Mu-like elements among bacterial genomes and their ORFs best-matches in the NCBI viral protein database. The degree of similarity and BLASTP expect values are shown in brackets. The genomic regions in Roseobacter are highlighted using green color. (XLS 5587 kb)

Additional file 3: Table S2. Hierarchical clustering of (pro)phages and prophage-like elements among 423 bacterial genomes in terms of gene content, which was based on SiliX homology clustering. Green represents the presence of gene. (XLS 291 kb)

Additional file 4: Table S3. Abundance and distribution of gpT genes of Mu-like phages in different Tara Oceans samples. (XLS 91 kb)

Additional file 5: Table S4. The predicted ORFs in vB_ThpS-P1 and vB_PeaS-P1 and their best-matches in the NCBI non-redundant (NR) protein database and NCBI viral protein database. The degree of similarity and BLASTP expect values are shown in brackets. (XLS 45 kb)

## Abbreviations

ANIb: Average nucleotide identity
ORFs: Open reading frames
PHAST: PHAge Search Tool, the prophage identification tool
RCB: Roseophages of Roseobacter clade bacteria
VLPs: Virus-like particles

## References

[1] Suttle CA (2005). Viruses in the sea. Nature.

[2] Suttle CA (2007). Marine viruses— major players in the global ecosystem. Nat Rev Microbiol.

[3] Dell’Anno A, Corinaldesi C, Danovaro R (2015). Virus decomposition provides an important contribution to benthic deep-sea ecosystem functioning. Proc Natl Acad Sci U S A.

[4] Mizuno CM, Rodriguez-Valera F, Kimes NE, Ghai R (2013). Expanding the marine virosphere using metagenomics. PLoS Genet.

[5] Brum JR, Sullivan MB (2015). Rising to the challenge: accelerated pace of discovery transforms marine virology. Nat Rev Microbiol.

[6] Sullivan MB, Waterbury JB, Chisholm SW (2003). Cyanophages infecting the oceanic cyanobacterium *Prochlorococcus*. Nature.

[7] Zhao Y, Temperton B, Thrash JC, Schwalbach MS, Vergin KL, Landry ZC (2013). Abundant SAR11 viruses in the ocean. Nature.

[8] Zhao Y, Wang K, Jiao N, Chen F (2009). Genome sequences of two novel phages infecting marine *Roseobacters*. Environ Microbiol.

[9] Wagner-Dobler I, Biebl H (2006). Environmental biology of the marine *Roseobacter* lineage. Annu Rev Microbiol.

[10] Brinkhoff T, Giebel HA, Simon M (2008). Diversity, ecology, and genomics of the *Roseobacter* clade: a short overview. Arch Microbiol.

[11] Rohwer F, Segall A, Steward G, Seguritan V, Breitbart M, Wolven F (2000). The complete genomic sequence of the marine phage Roseophage SIO1 shares homology with nonmarine phages. Limnol Oceanogr.

[12] Angly F, Youle M, Nosrat B, Srinagesh S, Rodriguez-Brito B, McNairnie P (2009). Genomic analysis of multiple Roseophage SIO1 strains. Environ Microbiol.

[13] Ankrah NY, Budinoff CR, Wilson WH, Wilhelm SW, Buchan A (2014). Genome sequence of the *Sulfitobacter* sp. strain 2047 infecting lytic phage ΦCB2047-B. Genome Announc..

[14] Ankrah NY, Budinoff CR, Wilson WH, Wilhelm SW, Buchan A (2014). Genome sequences of two temperate phages, ΦCB2047-A and ΦCB2047-C, infecting *Sulfitobacter* sp. strain 2047. Genome Announc.

[15] Huang S, Zhang Y, Chen F, Jiao N (2011). Complete genome sequence of a marine roseophage provides evidence into the evolution of gene transfer agents in *Alphaproteobacteria*. Virol J.

[16] Liang Y, Zhang Y, Zhou C, Chen Z, Yang S, Yan C (2016). Complete genome sequence of the siphovirus Roseophage RDJLΦ 2 infecting *Roseobacter denitrificans* OCh114. Mar Genomics.

[17] Kang I, Jang H, Oh HM, Cho JC (2012). Complete genome sequence of *Celeribacter* bacteriophage P12053L. J Virol.

[18] Chan J, Millard AD, Mann N, Schafer H (2014). Comparative genomics defines the core genome of the growing N4-like phage genus and identifies N4-like roseophage specific genes. Front Microbiol.

[19] Ji J, Zhang R, Jiao N (2015). Complete genome sequence of Roseophage vB_DshP-R1, which infects *Dinoroseobacter shibae* DFL12. Stand Genomic Sci.

[20] Cai L, Yang Y, Jiao N, Zhang R (2015). Complete genome sequence of vB_DshP-R2C, a N4-like lytic roseophage. Mar Genomics.

[21] Chen F, Wang K, Stewart J, Belas R (2006). Induction of multiple prophages from a marine bacterium: A genomic approach. Appl Environ Microbiol.

[22] Zhao Y, Wang K, Ackermann HW, Halden RU, Jiao N, Chen F (2010). Searching for a ‘hidden’ prophage in a marine bacterium. Appl Environ Microbiol.

[23] Engelhardt T, Sahlberg M, Cypionka H, Engelen B (2013). Biogeography of *Rhizobium radiobacter* and distribution of associated temperate phages in deep subseafloor sediments. ISME J..

[24] Engelhardt T, Kallmeyer J, Cypionka H, Engelen B (2014). High virus-to-cell ratios indicate ongoing production of viruses in deep subsurface sediments. ISME J..

[25] Engelhardt T, Orsi WD, Jørgensen BB (2015). Viral activities and life cycles in deep subseafloor sediments. Environ Microbiol Rep.

[26] Zhang X, Wang Y (2010). Genome analysis of deep-sea thermophilic phage D6E. Appl Environ Microbiol.

[27] Yoshida M, Yoshida-Takashima Y, Nunoura T, Takai K (2015). Genomic characterization of a temperate phage of the psychrotolerant deep-sea bacterium *Aurantimonas* sp. Extremophiles.

[28] Yoshida M, Yoshida-Takashima Y, Nunoura T, Takai K (2015). Identification and genomic analysis of temperate *Pseudomonas* bacteriophage PstS-1 from the Japan trench at a depth of 7,000 m. Res Microbiol.

[29] Li S, Tang K, Liu K, Jiao N (2015). *Thiobacimonas profunda* gen. nov., sp. nov., a member of the family *Rhodobacteraceae* isolated from the deep-sea water. Int J Syst Evol Microbiol.

[30] Lin Y, Tang K, Li S, Liu K, Sun J, Jiao N (2014). *Pelagibaca abyssi* sp. nov., of the family *Rhodobacteraceae*, isolated from deep-sea water. Antonie Van Leeuwenhoek.

[31] Tang K, Yang Y, Lin D, Li S, Zhou W, Han Y (2016). Genomic, physiologic, and proteomic insights into metabolic versatility in *Roseobacter* clade bacteria isolated from deep-sea water. Sci Rep.

[32] Morgan GJ, Hatfull GF, Casjens S, Hendrix RW (2002). Bacteriophage Mu genome sequence: analysis and comparison with Mu-like prophages in *Haemophilus, Neisseria* and *Deinococcus*. J Mol Biol.

[33] Zehr ES, Tabatabai LB, Bayles DO (2012). Genomic and proteomic characterization of SuMu, a Mu-like bacteriophage infecting Haemophilus parasuis. BMC Genomics.

[34] Wang P, Chu L, Guttman DS (2003). Complete sequence and evolutionary genomic analysis of the *Pseudomonas aeruginosa* transposable bacteriophage D3112. J Bacteriol.

[35] Braid MD, Silhavy JL, Kitts CL, Cano RJ, Howe MM (2004). Complete genomic sequence of bacteriophage B3, a Mu-like phage of *Pseudomonas aeruginosa*. J Bacteriol.

[36] Fogg PCM, Hynes AP, Digby E, Lang AS, Beatty JT (2011). Characterization of a newly discovered Mu-like bacteriophage, RcapMu, in *Rhodobacter capsulatus* strain SB1003. Virology.

[37] Zheng Q, Zhang R, Xu Y, White RA, Wang Y, Luo T (2014). A marine inducible prophage vB_CibM-P1 isolated from the aerobic anoxygenic phototrophic bacterium *Citromicrobium bathyomarinum* JL354. Sci Rep.

[38] Marie D, Brussaard CPD, Thyrhaug R, Bratbak G, Vaulot D (1999). Enumeration of marine viruses in culture and natural samples by flow cytometry. Appl Environ Microbiol.

[39] Brussaard CP (2004). Optimization of procedures for counting viruses by flow cytometry. Appl Environ Microbiol.

[40] Langmead B, Salzberg SL (2012). Fast gapped-read alignment with Bowtie 2. Nat Methods.

[41] Wiśniewski JR, Zougman A, Nagaraj N, Mann M (2009). Universal sample preparation method for proteome analysis. Nat Methods.

[42] Zerbino DR, Birney E (2008). Velvet: Algorithms for de novo short read assembly using de Bruijn graphs. Gen Res.

[43] Aziz RK, Bartels D, Best AA, DeJongh M, Disz T, Edwards RA (2008). The RAST Server: rapid annotations using subsystems technology. BMC Genomics.

[44] Medema MH, Takano E, Breitling R (2013). Detecting sequence homology at the gene cluster level with multigeneblast. Mol Biol Evol.

[45] Zhou Y, Liang Y, Lynch KH, Dennis JJ, Wishart DS (2011). PHAST: a fast phage search tool. Nucleic Acids Res.

[46] Miele V, Penel S, Duret L (2011). Ultra-fast sequence clustering from similarity networks with SiLiX. BMC Bioinforma.

[47] Tamura K, Stecher G, Peterson D, Filipski A, Kumar S (2013). MEGA6: Molecular evolutionary genetics analysis version 6.0. Mol Biol Evol.

[48] Richter M, Rosselló-Móra R (2009). Shifting the genomic gold standard for the prokaryotic species definition. Proc Natl Acad Sci U S A.

[49] Hunter S, Apweiler R, Attwood TK, Bairoch A, Bateman A, Binns D (2009). InterPro: the integrative protein signature database. Nucleic Acids Res.

[50] Sunagawa S, Coelho LP, Chaffron S, Kultima JR, Labadie K, Salazar G (2015). Structure and function of the global ocean microbiome. Science.

[51] Tang K, Jiao N, Liu K, Zhang Y, Li S (2012). Distribution and functions of TonB-dependent transporters in marine bacteria and environments: implications for dissolved organic matter utilization. PLoS One.

[52] Hurwitz BL, Sullivan MB (2013). The Pacific Ocean Virome (POV): A marine viral metagenomic dataset and associated protein clusters for quantitative viral ecology. PLoS One.

[53] Pau JH (2008). Prophages in marine bacteria: dangerous molecular time bombs or the key to survival in the seas?. ISME J.

[54] Denyes JM, Krell PJ, Manderville R, Ackermann HW, She YM, Kropinski AM (2014). The genome and proteome of *Serratia* bacteriophage η which forms unstable lysogens. Virol J.

[55] Cazares A, Mendoza-Hernández G, Guarneros G (2014). Core and accessory genome architecture in a group of *Pseudomonas aeruginosa* Mu-like phages. BMC Genomics.

[56] Ge J, Harshey RM (2008). Congruence of *in vivo* and *in vitro* insertion patterns in hot *E. coli* gene targets of transposable element Mu: opposing roles of MuB in target capture and integration. J Mol Biol.

[57] Ge J, Lou Z, Harshey RM (2010). Immunity of replicating Mu to self-integration: a novel mechanism employing MuB protein. Mob DNA.

[58] Hulo C, Masson P, Le Mercier P, Toussaint A (2015). A structured annotation frame for the transposable phages: A new proposed family ‘*Saltoviridae*’ within the *Caudovirales*. Virology.

[59] Reidl J, Mekalanos JJ (1995). Characterisation of *Vibrio cholerae* bacteriophage K139 and use of a novel mini-transposon to identify a phage-encoded virulence factor. Mol Microbiol.

[60] Heidelberg JF, Eisen JA, Nelson WC, Clayton RA, Gwinn ML, Dodson RJ (2000). DNA sequence of both chromosomes of the cholera pathogen *Vibrio cholerae*. Nature.

[61] Hayashi T, Makino K, Ohnishi M, Kurokawa K, Ishii K, Yokoyama K (2001). Complete genome sequence of enterohemorrhagic *Escherichia coli* O157:H7 and genomic comparison with a laboratory strain K-12. DNA Res.

[62] Summer EJ, Gonzalez CF, Carlisle T, Mebane LM, Cass AM, Savva CG (2004). *Burkholderia cenocepacia* phage BcepMu and a family of Mu-like phages encoding potential pathogenesis factors. J Mol Biol.

[63] Casjens SR (2005). Comparative genomics and evolution of the tailed bacteriophages. Curr Opin Microbiol.

[64] Casjens SR, Thuman-Commike PA (2011). Evolution of mosaically related tailed bacteriophage genomes seen through the lens of phage P22 virion assembly. Virology.

[65] Bobay LM, Touchon M, Rocha EPC (2014). Pervasive domestication of defective prophages by bacteria. Proc Natl Acad Sci U S A.

