# Supplementary material for: Genomic, proteomic and bioinformatic analysis of two temperate phages in Roseobacter clade bacteria isolated from the deep-sea water
Source: BMC Genomics. 2017 Jun 27;18:485. doi: 10.1186/s12864-017-3886-0 (PMC5488378; doi:10.1186/s12864-017-3886-0)
Supplement: Supplementary file 1 — Phylogenetic trees of the head modules proteins of (pro)phages. Maximum likelihood tree and neighbor-joining tree methods and bootstrap analysis (100 replicates) based on the alignment of the amino acid sequence of the I protein (A), the virion morphogenesis protein (B), the Mu-like phage F protein (C), the Mu-like phage gp29 protein (D), and the Mu-like phage gp28 protein (E) of (pro)phages. The numbers at the nodes indicate bootstrap probabilities of that particular branch of the maximum likelihood (above) and neighbor-joining (below) trees. Figure S2. Phylogenetic trees of the transcriptional regulator (A), transposase B (B), and transposase A (C) of the (pro)phages. The numbers at the nodes indicate the bootstrap probabilities of that particular branch of the maximum likelihood (above) and neighbor joining (below) trees. (PDF 191 kb) [file 12864_2017_3886_MOESM1_ESM.pdf]

Supplementary Figures

Genomic, Proteomic and Bioinformatic Analysis of Two Temperate Phages in *Roseobacter* Clade Bacteria Isolated from the Deep-sea Water

Kai Tang*, Dan Lin, Qiang Zheng, Keshao Liu, Yujie Yang, Yu Han, Nianzhi Jiao*

State Key Laboratory for Marine Environmental Science, Institute of Marine Microbes and Ecospheres, Xiamen University, Xiamen 361102, P. R. China

* Corresponding: tangkai@xmu.edu.cn and jiao@xmu.edu.cn

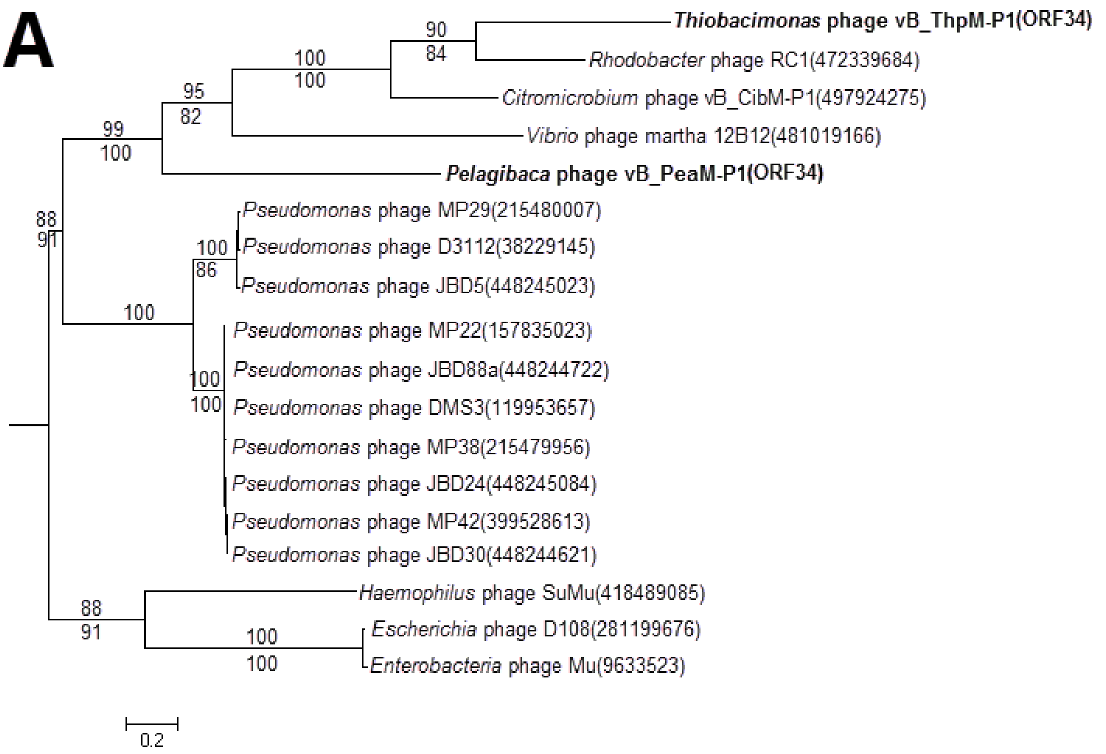
A
Thiobacimonas phage vB_ThpM-P1(ORF34)
Rhodobacter phage RC1(472339684)
Citromicrobium phage vB_CibM-P1(497924275)
Vibrio phage martha 12B12(481019166)
Pelagibaca phage vB_PeaM-P1(ORF34)
Pseudomonas phage MP29(215480007)
Pseudomonas phage D3112(38229145)
Pseudomonas phage JBD5(448245023)
Pseudomonas phage MP22(157835023)
Pseudomonas phage JBD88a(448244722)
Pseudomonas phage DMS3(119953657)
Pseudomonas phage MP38(215479956)
Pseudomonas phage JBD24(448245084)
Pseudomonas phage MP42(399528613)
Pseudomonas phage JBD30(448244621)
Haemophilus phage SuMu(418489085)
Escherichia phage D108(281199676)
Enterobacteria phage Mu(9633523)
0.2

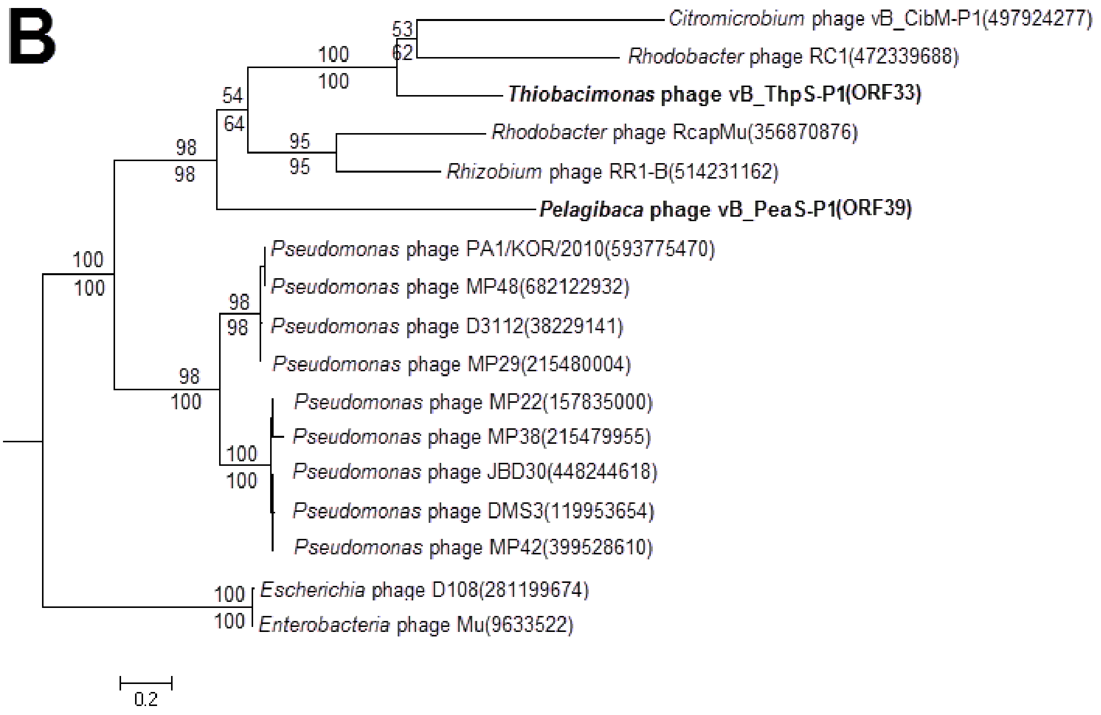
B
Citromicrobium phage vB_CibM-P1(497924277)
Rhodobacter phage RC1(472339688)
Thiobacimonas phage vB_ThpS-P1(ORF33)
Rhodobacter phage RcapMu(356870876)
Rhizobium phage RR1-B(514231162)
Pelagibaca phage vB_PeaS-P1(ORF39)
Pseudomonas phage PA1/KOR/2010(593775470)
Pseudomonas phage MP48(682122932)
Pseudomonas phage D3112(38229141)
Pseudomonas phage MP29(215480004)
Pseudomonas phage MP22(157835000)
Pseudomonas phage MP38(215479955)
Pseudomonas phage JBD30(448244618)
Pseudomonas phage DMS3(119953654)
Pseudomonas phage MP42(399528610)
Escherichia phage D108(281199674)
Enterobacteria phage Mu(9633522)
0.2

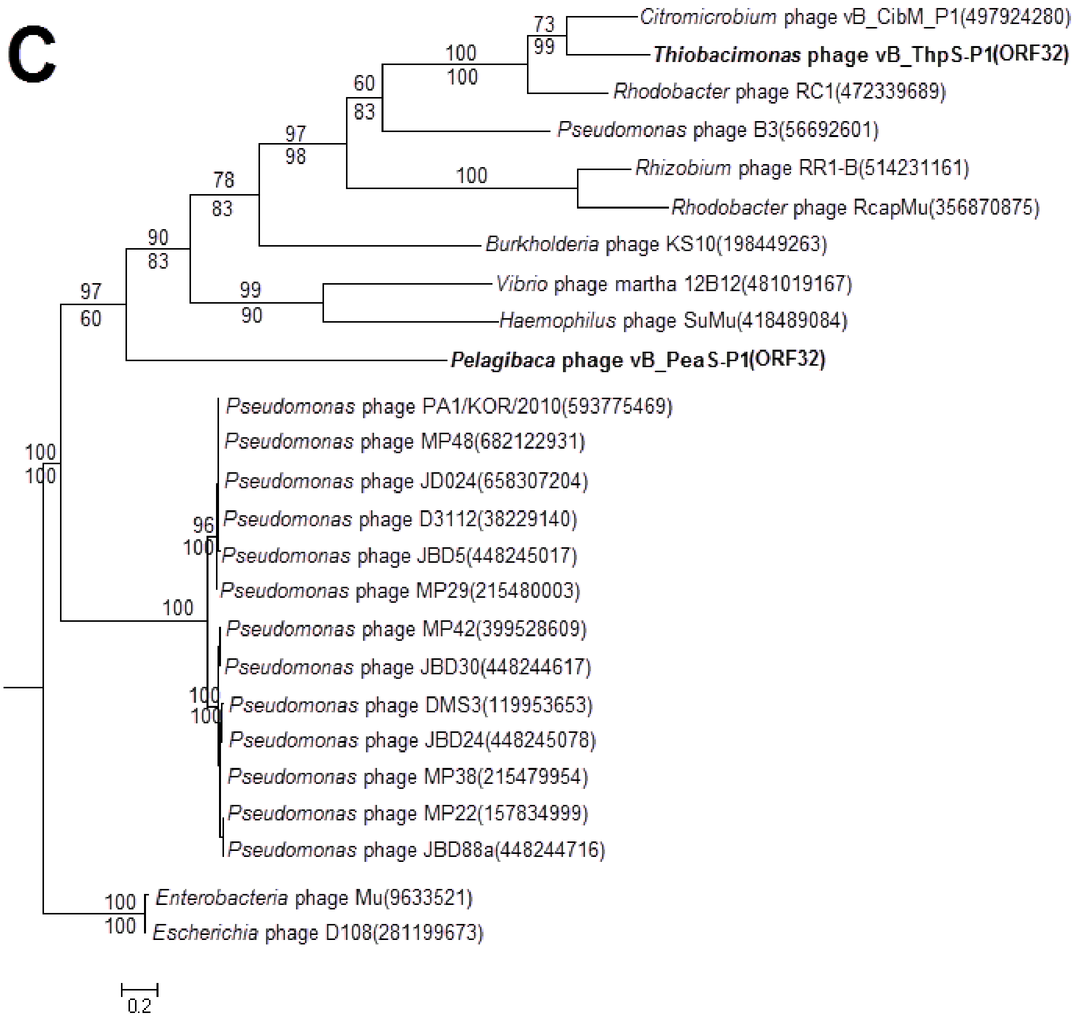
C
Citromicrobium phage vB_CibM_P1(497924280)
Thiobacimonas phage vB_ThpS-P1(ORF32)
Rhodobacter phage RC1(472339689)
Pseudomonas phage B3(56692601)
Rhizobium phage RR1-B(514231161)
Rhodobacter phage RcapMu(356870875)
Burkholderia phage KS10(198449263)
Vibrio phage martha 12B12(481019167)
Haemophilus phage SuMu(418489084)
Pelagibaca phage vB_PeaS-P1(ORF32)
Pseudomonas phage PA1/KOR/2010(593775469)
Pseudomonas phage MP48(682122931)
Pseudomonas phage JD024(658307204)
Pseudomonas phage D3112(38229140)
Pseudomonas phage JBD5(448245017)
Pseudomonas phage MP29(215480003)
Pseudomonas phage MP42(399528609)
Pseudomonas phage JBD30(448244617)
Pseudomonas phage DMS3(119953653)
Pseudomonas phage JBD24(448245078)
Pseudomonas phage MP38(215479954)
Pseudomonas phage MP22(157834999)
Pseudomonas phage JBD88a(448244716)
Enterobacteria phage Mu(9633521)
Escherichia phage D108(281199673)
0.2

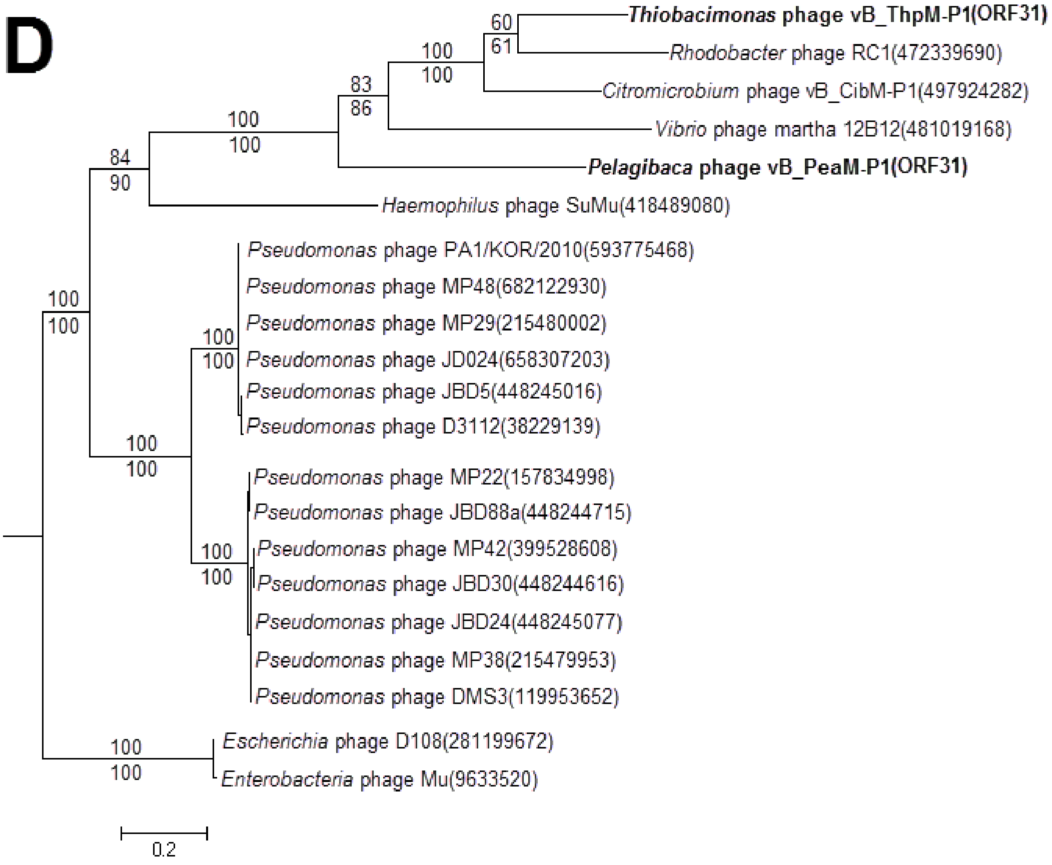
D
Thiobacimonas phage vB_ThpM-P1(ORF31)
Rhodobacter phage RC1(472339690)
Citromicrobium phage vB_CibM-P1(497924282)
Vibrio phage martha 12B12(481019168)
Pelagibaca phage vB_PeaM-P1(ORF31)
Haemophilus phage SuMu(418489080)
Pseudomonas phage PA1/KOR/2010(593775468)
Pseudomonas phage MP48(682122930)
Pseudomonas phage MP29(215480002)
Pseudomonas phage JD024(658307203)
Pseudomonas phage JBD5(448245016)
Pseudomonas phage D3112(38229139)
Pseudomonas phage MP22(157834998)
Pseudomonas phage JBD88a(448244715)
Pseudomonas phage MP42(399528608)
Pseudomonas phage JBD30(448244616)
Pseudomonas phage JBD24(448245077)
Pseudomonas phage MP38(215479953)
Pseudomonas phage DMS3(119953652)
Escherichia phage D108(281199672)
Enterobacteria phage Mu(9633520)
0.2

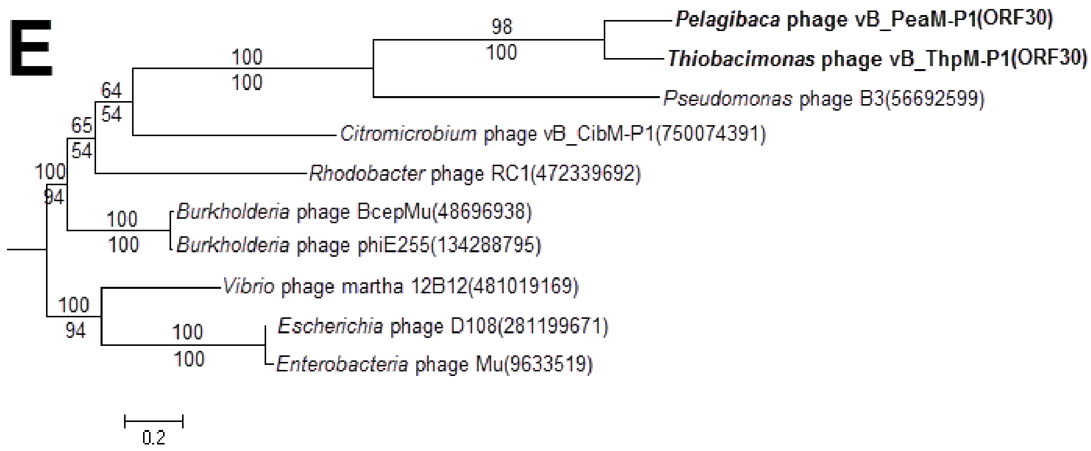

Figure S1. Phylogenetic trees of the head modules proteins of (pro)phages. Maximum likelihood tree and neighbor-joining tree methods and bootstrap analysis (100 replicates) based on the alignment of the amino acid sequence of the I protein (A), the virion morphogenesis protein (B), the Mu-like phage F protein (C), the Mu-like phage gp29 protein (D), and the Mu-like phage gp28 protein (E) of (pro)phages. The numbers at the nodes indicate bootstrap probabilities of that particular branch of the maximum likelihood (above) and neighbor-joining (below) trees.

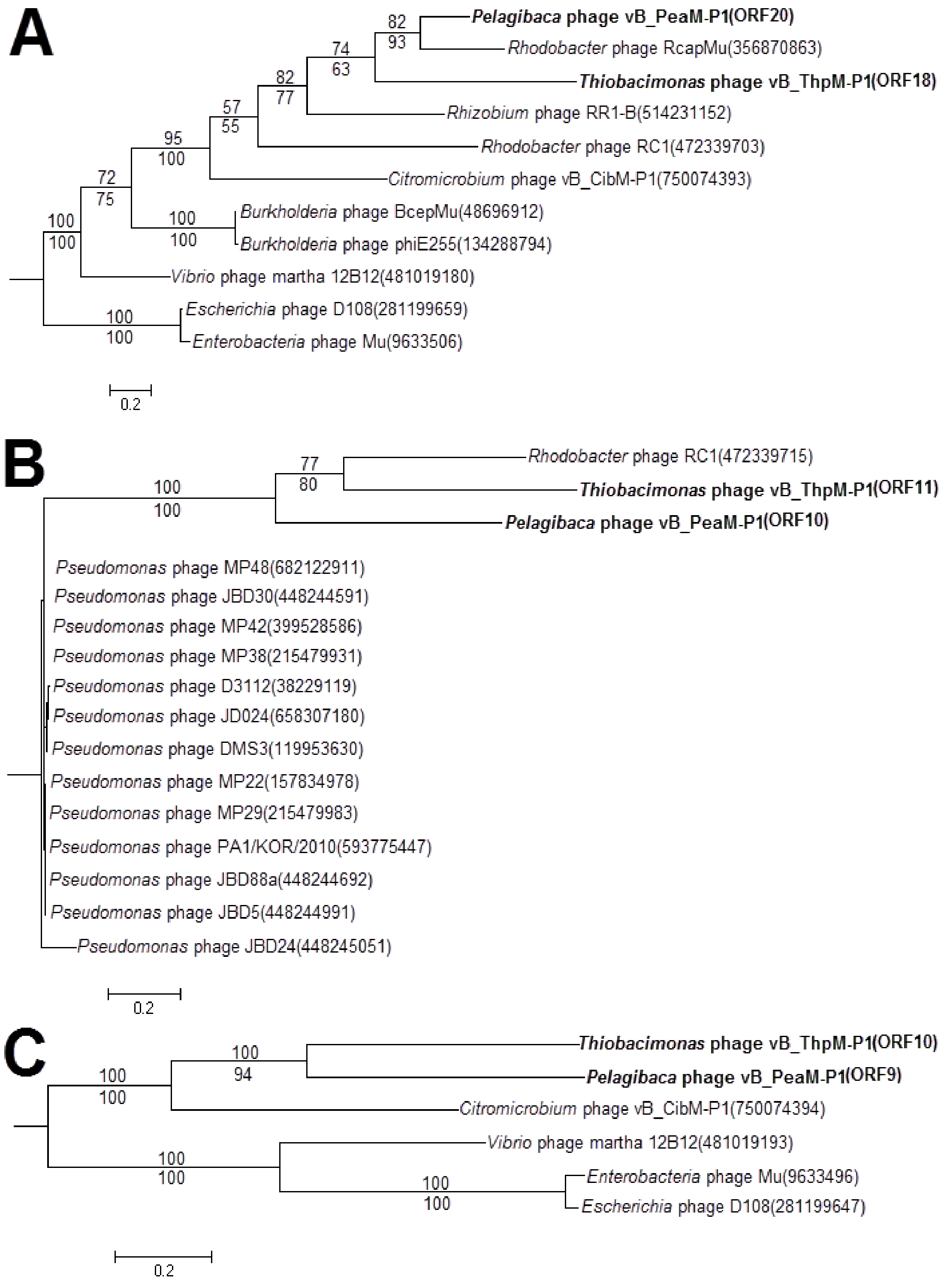

Figure S2. Phylogenetic trees of the transcriptional regulator (A), transposase B (B), and transposase A (C) of the (pro)phages. The numbers at the nodes indicate the bootstrap probabilities of that particular branch of the maximum likelihood (above) and neighbor joining (below) trees.
